# Prevalence of chronic diseases among United Arab Emirates University students: cross-sectional study

**DOI:** 10.1093/eurpub/ckac131.142

**Published:** 2022-10-25

**Authors:** MM Bashir, M Alshamsi, S Almahrooqi, T Alyammahi, W Alhammadi, S Alshehhi, H Alhosani, F Alhammadi, F Al-Maskari

**Affiliations:** 1 Institute of Public Health, United Arab Emirates University, Al Ain, United Arab Emirates; 2 College of Medicine and Health Sciences, United Arab Emirates University, Al Ain, United Arab Emirates; 3 Zayed Centre for Health Sciences, United Arab Emirates University, Al Ain, United Arab Emirates

## Abstract

**Background:**

Chronic disease burden is increasing globally. In Arab Gulf Countries, the burden has increased exponentially over the past five decades due to rapid economic growth and urbanization. In the United Arab Emirates (UAE), chronic diseases are the leading cause of mortality and economic burden, hence, there is need to explore their patterns for targeted interventions. Studies among university students in Europe and the United States show chronic diseases prevalence ranging from 16.5% to 30.0%, respectively. To our knowledge, this is the first study in the Gulf region to assess multiple chronic diseases among university students. Our study describes the prevalence and patterns of multiple chronic diseases among UAE University (UAEU) students.

**Methods:**

We conducted a descriptive cross-sectional study among UAEU students ≥18years from July to October 2021. Online questionnaire was used to collect data. Self-reported chronic diseases were described and compared between male and female students using chi-square and t tests. Other students’ characteristics were also explored. All analyses were conducted using STATA statistical software.

**Results:**

902 students participated in the study with mean age of 21.9±5.2yrs. 79.8% were females. 80.7% were undergraduates. The prevalence of self-reported chronic diseases was 23.0%. Obesity, Diabetes and Asthma/Allergies were the commonest (12.5%, 4.2% & 3.2%, respectively). 34.8% of the students were either overweight or obese. Overall chronic disease prevalence was similar between males and females [27.5% vs 21.8%, 0.104] though it was significantly higher among postgraduates, students who are older, married and have family history of diabetes. 4.7% of the students reported 2 or more chronic diseases.

**Conclusions:**

Our study showed that more than 1 in 5 of the students reported at least one chronic disease. This shows the need for proactive chronic disease screening and prevention programs to meet the health needs of the students.

**Key messages:**

• Prevalence of chronic diseases (Diabetes, Prediabetes, Obesity, Hypertension, Asthma/Allergies, Lipid disorders, Thyroid disorders, GI disorders, and CVDs) was high among the university students.

• Universities should commit to researching students’ health and creating targeted health policies and interventions, as chronic diseases have direct and indirect negative impact on students’ education.

